# Facilitators and Barriers of Using an Artificial Intelligence Agent in Chronic Disease Management: A Normalization Process Theory-Guided Qualitative Study of Older Patients with COPD

**DOI:** 10.3390/healthcare14020268

**Published:** 2026-01-21

**Authors:** Shiya Cui, Shilei Wang, Jingyi Deng, Ruiyang Jia, Yuyu Jiang

**Affiliations:** 1 Research Office of Chronic Disease Management and Rehabilitation, Department of Nursing, Wuxi School of Medicine, Jiangnan University, No. 1800 Lihu Avenue, Wuxi 214122, China; 6232807002@stu.jiangnan.edu.cn (S.C.); 6242807022@stu.jiangnan.edu.cn (S.W.); 2022211401039@stu.hznu.edu.cn (J.D.); 19506285569@163.com (R.J.); 2 School of Public Health and Nursing, Hangzhou Normal University, Hangzhou 311121, China; 3 School of Medicine, Liaocheng University, Liaocheng 252000, China

**Keywords:** artificial intelligence, older adults, chronic obstructive pulmonary disease, chronic disease management, qualitative study

## Abstract

Objectives: This study aims to explore the facilitators and barriers in the process of using AI agents for disease management in older COPD patients. Methods: Based on the normalization process theory, a descriptive qualitative study was used to conduct semi-structured interviews with 28 older patients with COPD recruited from June to August 2025 in a Class A tertiary hospital in Wuxi, Jiangsu Province. Results: A total of 28 interviews were conducted. Four themes (Coherence, Cognitive Participation, Collective Action, Reflexive Monitoring), nine subthemes (recognition of intelligent technology;supported by policy discourse and the background of national-level projects; the creation of a family atmosphere; recommendations from HCPs; relief and social connection; new “doctor”–patient relationship and communication; eliminate the burden and return to life; benefit and value perception; right self-decision by AI) in facilitators and nine subthemes (privacy conflicts and trust deficiency; blurred boundaries of human–machine responsibility and authority; non-high-quality services are chosen reluctantly; technical anxiety; lack of motivation for continued engagement; extra burden; limitations of the physical environment; human–machine dialogue frustration; a sense of uncertainty about the future of AI) in barriers were extracted. Conclusions: This study identified key factors influencing the use of AI agents in chronic disease management in older patients with COPD. The results provide directions for improving the implementation and sustainable use of AI health technologies.

## 1. Introduction

Chronic obstructive pulmonary disease (COPD) is a chronic respiratory condition characterized by high prevalence, high disability rates, and high mortality [1]. It imposes a significant burden on healthcare systems worldwide. The prevention, treatment, and management of COPD have become critical global public health issues [2]. Pulmonary rehabilitation (PR), as a core component of comprehensive COPD management, has been demonstrated to significantly improve patients’ exercise tolerance, alleviate breathlessness, and enhance quality of life [3]. However, multiple studies indicated that COPD patients generally exhibit low rehabilitation adherence in chronic disease management [4,5]. Traditional rehabilitation management models are limited by insufficient medical resources, low service coverage, and inadequate doctor–patient communication. This makes it difficult to meet older patients’ requirements for continuous, personalized, and frequent rehabilitation guidance throughout the entire disease process [6]. Therefore, exploring innovative management strategies is crucial for enhancing the health outcomes of older patients with COPD.

The advancement of digital health has opened new perspectives for chronic disease management. It achieved broader population coverage and sustained health management by enhancing the accessibility and cost-effectiveness of healthcare services [7]. In recent years, the research focus of digital health has changed. It is progressively moving away from traditional eHealth and mHealth towards intelligent solutions. Among these, the rapid advancement of artificial intelligence (AI) has been particularly noteworthy. AI has been extensively applied in disease prevention [8], risk prediction [9], clinical decision support [10], and rehabilitation guidance [11]. Their potential in chronic disease management is obvious. A systematic review indicated that the effectiveness of AI chatbots in managing chronic conditions such as cancer, hypertension, and diabetes has been demonstrated [12]. The latest evidence indicates that the application of AI in the field of COPD is primarily focused on early disease screening and diagnosis, the identification of COPD exacerbation, optimization of diagnosis and treatment, disease monitoring, assessment of prognosis, etc. [13,14,15].

Although the efficacy of AI applications in chronic disease management has been demonstrated, their real-world intervention outcomes remain significantly influenced by patient engagement and sustained usage. Previous studies have highlighted that the practical application of e-health technology is often hindered by multiple factors at the organizational, technological, and user levels. There is a significant gap between the implementation effect and the theoretical expectation [16,17,18]. This indicates that it is crucial to identify the barriers or facilitators for older patients with chronic diseases to use AI for disease management. This will determine the success or failure of applying AI in chronic disease management. Current research predominantly focuses on validating the technical performance and clinical efficacy of AI, while paying insufficient attention to the subjective experiences and attitudes. Although a small number of qualitative studies have explored the user experience of AI in healthcare within the fields of oncology and cardiovascular disease [19,20], relevant evidence concerning COPD patients remains severely lacking. Additionally, the common problem of technological anxiety among older people with COPD affects the application of AI in disease management [21]. Therefore, it is necessary to systematically explore the experiences and potential impacts of using AI among older patients with COPD. This can help researchers gain a deeper understanding of the current application of AI in chronic disease management.

In conclusion, the use of AI for disease management in older patients is influenced by multiple factors. Employing a theoretical framework offers a structured approach to identify and analyze these influences [22]. Consequently, conducting qualitative research within such a framework is essential to gain a deeper understanding of the barriers and facilitators affecting the adoption of AI technology in chronic disease management for older adults. Normalization Process Theory (NPT) is one of the implementation science theories [23]. It includes four dimensions, namely, “Coherence”, “Cognitive Participation”, “Collective Action”, and “Reflexive Monitoring”. This theory provides a systematic explanation of how complex interventions become embedded, normalized, and sustained within clinical practice.

This study is qualitative research from the perspective of older people with COPD. It aims to deeply explore the real experiences, barriers, and facilitators of using AI agents in chronic disease management. The interviewees are from a randomized controlled trial (ChiCTR2500096384). The intervention tool in this randomized controlled trial is an AI agent (hereinafter referred to as AI) designed specifically to help COPD patients enhance their rehabilitation motivation and self-efficacy, so as to improve their rehabilitation adherence. The findings of this research can hopefully promote the effective translation of AI agents into the chronic disease management of older adults.

## 2. Materials and Methods

### 2.1. Design

This study was a descriptive qualitative study to explore the facilitators and barriers of using AI agents for disease management in older COPD patients. It was nested in an ongoing RCT trial (Clinical trial Registration number, ChiCTR2500096384). This study was conducted in accordance with the Declaration of Helsinki and followed the Consolidated Criteria for Reporting Qualitative Research (COREQ) guidelines for data reporting (see Supplementary Materials S1) [24]. Ethical approval was obtained from the Medical Ethics Committee of Jiangnan University (JNU202409RB0014).

### 2.2. Participants and Recruitment

The purposive sampling method was used to recruit older COPD patients with different ages, genders, and education levels from the intervention group of previous RCTs from June to August 2025 from a hospital in Wuxi, Jiangsu Province, following the principle of data saturation. Inclusion criteria: (1) patients who had participated in the intervention group of the above RCT, that is, patients with disease management experience using AI agents; (2) diagnosed with COPD by a municipal medical institution (in stable phase, FEV_1/_FVC < 70%, FEV_1_ < 80%) [25]; (3) age ≥ 65; (4) able to communicate in Chinese. Exclusion criteria: patients with cognitive or communication impairments, or those whose condition has worsened and are unable to complete the interview.

### 2.3. Research Framework

NPT was originally developed by scholars May et al. as a theoretical model to help clinicians and nurses understand and evaluate the factors that promote or hinder the integration of healthcare interventions into routine practice [26,27,28]. Murray et al. proposed that the dynamic interaction among the four dimensions of NPT: coherence, cognitive participation, collective action, and reflexive monitoring can effectively explain the implementation process [23,29]. Additionally, NPT focuses on how new technologies are understood, accepted, and integrated into daily behavioral routines, emphasizing individuals’ cognition, participation, and actions during this process. It has been widely used in the feasibility and process evaluation of interventions in medical services, public health, and other fields [30,31]. Therefore, to ensure systematic and theoretical support of this study, NPT will be used as the analytical framework based on the research objectives.

Guided by the NPT theory, we developed an interview outline focusing on the facilitators and barriers of using AI agents for disease management in older COPD patients. A pre-interview was conducted with the first two patients before the formal interview, and the interview outline did not require further modification after discussion by the research group. Table 1 presents the theoretical mapping table of the interview outline.

### 2.4. Data Collection

The research team facilitated face-to-face communication with the patients. After establishing a positive rapport, an overview including research objectives, research content, precautions, confidentiality, voluntary participation, risks, and benefits was introduced to the patients. The location and duration were determined after obtaining both verbal and written informed consent from the patients. A self-designed questionnaire was used to collect general information from the patients, including age, gender, marital status, education level, monthly income, smoking status, disease duration, GOLD classification, and the number of hospitalizations in the past year.

Researchers received relevant training and conducted many simulated interviews before the formal interview. To ensure data accuracy and consistency, all interviews were conducted by C.S.Y., the first author of this study. Each interview took place in the patient’s home in a quiet environment, without third-party personnel. The interviews were face-to-face and semi-structured. Each interview lasted between 30 and 50 min, and each patient was interviewed only once. In order to ensure the quality of the interview, the whole interview was recorded with a voice recorder, and the expressions and movements of the patients were observed. If necessary, the order of questions could be adjusted, allowing for timely responses and supplementary questions. Researchers avoided using instructive or suggestive language to ensure that the interviews remained focused on the relevant topics. Data collection and analysis were performed simultaneously. During the interview process, the research team coded each interview transcript and performed thematic analysis, continuously monitoring the emergence of new themes. After the 26th interview, the research team considered that the themes had stabilized, and to verify the reliability of this judgment, two additional interviews were conducted, which resulted in no new coding or themes. After analyzing data from 28 interviews, no new themes emerged. The research team therefore reached a consensus that the data had reached saturation, so further data collection was stopped.

### 2.5. Data Analysis

Within 24 h of the interview, the researcher (C.S.Y.) conducted a verbatim transcription of the recording, cross-referenced with field notes for verification. The accuracy of the transcribed text was verified by researcher W.S.L. through repeated careful listening to the recording and thorough review of the transcript. The transcripts were returned to the participants for confirmation. Thematic analysis was employed to examine the data, utilizing the four core dimensions of the NPT model as the initial thematic framework. Data management was conducted using the qualitative analysis software NVivo 12 Plus. To ensure the reliability of the coding, investigators C.S.Y. and J.Y.Y. independently preliminarily coded the interview transcripts during the analysis process. They repeatedly read, familiarized, and immersed themselves in the text materials, and marked the key statements, preliminary feelings, and confusion. Following this preliminary immersive analysis, an initial analytical framework was established based on the aforementioned dimensions, with the four core dimensions serving as themes for systematic coding. Subsequently, materials under each theme were grouped, organized, and categorized into subthemes according to similarity and relevance. In case of coding discrepancies, the research team discussed together until a consensus was reached, and a third researcher was invited to participate in arbitration if necessary. Finally, the theme framework was determined after collective review, discussion, and examination by the research team. Statistical consistency indicators such as kappa coefficient were not calculated in this study, but the transparency of the coding process and the credibility of the analysis results were ensured by independent coding, team discussion, and collective review.

## 3. Results

### 3.1. Demographic Characteristics of Participants

Finally, twenty-eight older patients with COPD were enrolled in this study, including twenty-five males and three females. Each participant was interviewed one-on-one and lasted from 30 to 50 min without a second interview. The basic characteristics of the participants are shown in Table 2.

### 3.2. Thematic Analysis

**Figure 1:**
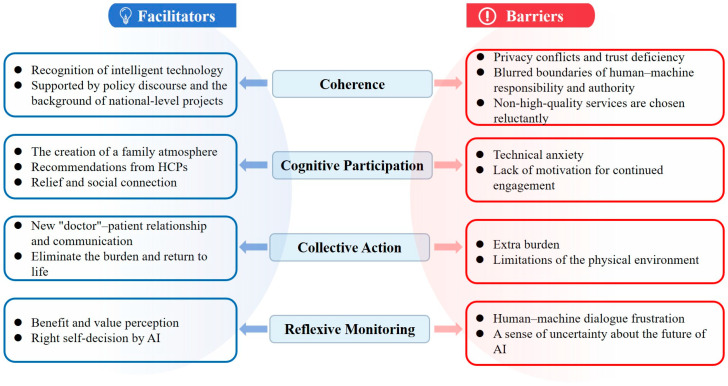
Facilitators and barriers to AI adoption based on NPT (The blue sections represent facilitators and the red sections represent barriers).

According to the NPT, the barriers and facilitators of using AI agents for disease management in older COPD patients were identified and classified. Using the four dimensions of the theory as themes, we found nine subthemes of facilitators (see Table 3 and Figure 1) and nine subthemes of barriers (see Table 4 and Figure 1). Supplementary Materials S2 presents examples of the theme generation process.

#### 3.2.1. Facilitators

Theme 1: Coherence
Recognition of intelligent technology

The interviews found that mass media and intelligent interactive devices promoted patients’ understanding, perception, and use of AI. The AI use experience obtained by patients in non-medical scenarios allows patients to recognize AI technology, and it lays a cognitive foundation for patients to accept and apply this technology in the field of health management.

*I have known about AI for a long time. It said on TV that it can win the world championship in chess, which I think is really impressive*.

(P4)

*AI is really convenient, just like my Tmall genie or Siri. I just speak, and it answers immediately*.

(P4)

*Didn’t the TV say we already have smart hospitals? I think that’s pretty great*.

(P19)

Supported by policy discourse and the background of national-level projects

Government-sponsored, non-commercial AI provided by public institutions at the national level have credibility. Some participants indicated that policy opinion made them willing to participate in the program. Patients also mentioned that the endorsement of national programs made them feel safe and reliable. Aside from this, some patients reported that they preferred to use non-commercial AI provided by professional scientific research institutions.

*I’ve seen many documents and news reports saying that we should promote AI technologies, and it seems the government is quite supportive. So I wanted to give it a try*.

(P20)

*The project is funded by the state, so it’s very secure, and I don’t worry about security and privacy*.

(P15)

*This is not a commercial software downloaded from the Internet, but a public welfare AI tool provided by professional scientific research institutions... I’d rather use a tool like this*.

(P16)

Theme 2: Cognitive Participation
The creation of a family atmosphere

Enjoying the intimacy of family was the motivation for patients to use AI. Some patients said that their willingness to use AI usually comes after observing their friends and family using them. Several participants mentioned that they attempted to use it with the supervision and assistance of their children. A few others shared that they experimented with AI tools to add to the conversation during family gatherings.

*Since I saw my grandchildren using AI, I wanted to try it too*.

(P10)

*I didn’t want to use it, but my kids pushed me to try it and helped me download Deepseek on my phone*.

(P2)

*My kids and my wife were talking about AI, so I immediately asked my kids to help me with it, so I could talk about it next time*.

(P9)

Recommendations from HCPs

Authority effect is an important motivation for patients to use AI. Several participants mentioned that they did not really trust AI. However, they felt that recommendations from healthcare professionals (HCPs) could alleviate some of their concerns regarding technology and privacy risks. Some respondents believed that only the endorsement of their personal attending physician or nurse could convince them of AI’s reliability, and they indicated that they did not consider the risks of AI.

*My attending doctor personally helped me register, and because of trusted him, I chose to trust that there would be no risk in using AI and that my privacy would not be compromised*.

(P23)

*The nurse in charge of me said I could keep using this tool, so I figured it must be legitimate and reliable. Others recommended what I did not believe. I did not think so much about the risks of AI*.

(P6)

Theme 3: Collective Action
Relief and social connection

AI provides psychological support for the disease management process of patients with chronic diseases to help patients relieve loneliness and stigma in group identity, so that respondents feel that they are only a drop in the ocean of many patients, and they feel relieved. Furthermore, AI allows them to see a colorful world through information connection, maintaining the patient’s interaction with society.

*I used to think that I was the only one with this disease, but now I know that there are many people like me, and I don’t feel lonely*.

(P16)

*... I’ve come to realize that I’m just a drop in the ocean*.

(P20)

*AI is so powerful, just like a kaleidoscope, let me see what I did not know before, I feel that I am not out of the world*.

(P1)

New “doctor”–patient relationship and communication

AI empowers patients and subverts the traditional authority model between doctors and patients. Some participants noted that during AI-led disease management, they could interact with the AI freely, including asking questions, sharing complaints, and even venting their temper. This unexpected new “doctor”–patient relationship breaks the traditional model, making patients feel that they have shifted from passive followers of HCPs to individuals with their own agency and control. In addition, AI improves the efficiency and quality of diagnosis and treatment. Some respondents believed that since the AI recorded every detail of their lives, doctors could quickly access a comprehensive picture of their condition, enabling them to offer the best treatment plan.

*I used to be overcautious when seeing a doctor, and I didn’t dare to say much to the doctor. This specialized AI is not a real person. I can talk freely, express opinions, say what I think, and even lose my temper, and feel like I am the master of it*.

(P19)

*I never thought that doctor-patient communication could be like this*...

(P11)

*With the AI report, the doctor could quickly and fully understand me, and I could explain my symptoms and needs more clearly. I believe this leads to the best treatment plan*.

(P15)

Eliminate and return to life

The technological advantage of AI in transcending temporal and spatial constraints has reshaped emotional relationships in family caregiving. Previously, respondents often felt pressure and guilt regarding their children’s care. The continuous and clear health data presented by AI has become a reassurance for both children and their parents. Additionally, AI empowers family members and enhances the professional competence of non-specialized caregivers. Some respondents said that with the prompt and accurate guidance of AI, family members have changed from a layman to a half-expert. Furthermore, some respondents mentioned that although AI did not change the disease itself, it skillfully returned the focus of life from the illness to life itself.

*In the past, my daughter kept asking on the phone, “Dad, how are you?” The more she cared, the more pressure and indebted I felt. Now she can see my data directly on her phone—it reassures her, and it puts my mind at ease too*.

(P16)

*In the past, my wife took care of me blindly, like a powerless outsider. Now AI often gives us practical rehabilitation advice, so that my wife is like a half-expert, easy to handle and targeted*.

(P1)

*Every conversation at home was about my illness previously. Now, AI has helped me set clear goals, which naturally shifted our focus from the disease to how to accomplish goals. The topic of conversation at home has finally returned to daily necessities and family life*.

(P20)

Theme 4: Reflexive Monitoring
Benefit and value perception

Most participants not only recognized the functional value of AI, but also perceived its comprehensive use value at the psychological and social levels. Some mentioned that their daily activities had expanded, their lives had become richer, and they were full of hope for the future after using AI agents for disease management. Some people also said that AI felt like a tireless, real-time companion, caring for them like a family member, and that it brought them good spirits and a sense of hope.

*After using this AI, I used to be able to stay at home, but now I can go out for a stroll and buy food. I feel that I have not insisted on exercise in vain, and I have hope for life*.

(P11)

*It always answers my questions and cares about me like my friends and family... Every time I finish talking about my pain with it, my mood is much better, and I feel that the future is full of hope. I think the value of AI in the future is immeasurable*.

(P23)

Right self-decision by AI

Many respondents said they would stick to their initial decision to choose AI. AI enables them to shift from fear of disease to taking control of their health, experiencing unexpected benefits, and achieving visible progress.

*In the past, whenever I wheezed, I would worry and be afraid. Now, AI has helped me to make risk warnings and plans early, and I was able to cope with them with ease and prepare in advance. I couldn’t appreciate the feeling of having my health in my own hands, which was the most valuable change brought by this decision*.

(P11)

*Surprisingly, my decision not only stabilized my condition but also alleviated my family’s anxiety*.

(P1)

*A compliment, an extra few meters of walking... The growth that AI has brought to me is the most powerful proof of my decision*.

(P23)

#### 3.2.2. Barriers

Theme 1: Coherence
Privacy conflicts and trust deficiency

Patients expressed concerns about privacy breaches, feeling as 24-h-monitored guinea pigs and that their data could be exploited for commercial gain, making them reluctant to accept long-term AI-based disease management. Some patients lack trust in AI. They viewed AI as merely a gimmick by hospitals or tech companies to attract people to pay and thus found it hard to entrust their health or even their lives to something intangible.

*The thought of my physical data being constantly recorded, uploaded, and analyzed makes me feel like a lab rat under 24-h surveillance, which makes me extremely uneasy and resistant. I’m also concerned that my data may be used for commercial purposes*.

(P5)

*I think it’s probably just another gimmick for a hospital or a tech company. How could I entrust my health, or even my life, to an AI that I can neither see nor touch*?

(P14)

Blurred boundaries of human–machine responsibility and authority

The interviews revealed that patients lack a clear understanding of the role orientation, scope of authority, and decision boundaries of AI. Some patients indicated that they are unclear about the relationship between AI, patients, and doctors, often having vague ideas about who is the leader, who is the assistant, or who is the collaborator. Additionally, there was confusion about what AI can and cannot do. Some patients also worried that AI may conflict with doctors’ decisions or that when AI makes mistakes, it is unclear who should take responsibility.

*I can’t tell whether it is my assistant or my leader. I also don’t know whether I should listen to the doctor or the AI when their advice differs*.

(P24)

*But it was like a black box, and I didn’t know what it could and couldn’t do*.

(P12)

*I’m worried that if something goes wrong with it, who will be responsible*?

(P18)

Non-high-quality services are chosen reluctantly

Some patients expressed resistance to technological substitution. They believed that high-quality health care is embodied by a “real man” who possesses professional experience and can connect emotionally with patients. They viewed the use of machine services as a last resort rather than the ideal form of quality health care.

*An experienced doctor who shakes my hand and looks me in the eye will make me feel warm, which is high-quality service*.

(P2)

*It is said that handmade is luxury and high quality. I hope that the service provided by human beings, machine service is a kind of helpless choice*.

(P8)

Theme 2: Cognitive Participation
Technical anxiety

Older and less educated participants mentioned more that when they first encountered AI, their unfamiliarity with digital technology and fear of making mistakes led them to experience technical barriers and a sense of caution. Later, as they become more proficient in using AI, self-doubt may also occur.

*Faced with these new things, I was like a schoolboy, and I didn’t know where to start*.

(P12)

*I’m afraid what if I make a mistake... I’ve been using it very cautiously*.

(P13)

*Now I can follow the steps, but sometimes I still wonder: Am I using the right one? If one step is wrong, will the AI be wrong*?

(P18)

Lack of motivation for continued engagement

The lack of motivation of patients mainly stems from the lack of freshness and the lack of immediate perceived positive feedback. Some patients reported that their motivation to continue participating decreased significantly once the novelty of AI wore off. Initially, they would check in and record health data promptly, but as tasks and reminders became routine, their interest and motivation faded. Moreover, the lack of immediate positive feedback or noticeable improvements in their health or daily activities (such as household chores) makes it more challenging to remain committed.

*At first, I checked in diligently every day, but after a month, the constant reminders felt meaningless, so I gradually got lazy and lost the motivation to use it*.

(P24)

*I follow the AI’s instructions and keep exercising, but I don’t see any improvement in myself, and no one tells me I’m doing well, so I start to feel discouraged*.

(P5)

*I have to do housework and accompany my children every day, and I really can’t stick to using AI*.

(P12)

Theme 3: Collective Action
Extra burden

The inconvenient operation of AI is the source of a burden for patients. Some patients mentioned that complex login verification, trivial data entry, high-frequency clocking reminders, and other system operations were unnecessary. In addition, the lack of humanistic care in the interactive way brings psychological discomfort to patients. Some patients thought that the repeated reminders of AI brought strong implications of disease identity to patients and psychological stress.

*Every time you open it, you have to verify, log in, etc., which is too complicated... I have to fill in all kinds of questionnaires and cumbersome data, which is annoying, and I think it is completely unnecessary*.

(P14)

*Every time I clocked in, every task reminder, it was like reminding me that I was a patient and brought me a great burden*.

(P17)

Limitations of the physical environment

Respondents mentioned that the use of AI is significantly limited by network conditions, device performance, and application stability. Unstable network and old equipment were the most common obstacles mentioned by patients, which would lead to a decrease in patients’ willingness to use it; it even weakened the enthusiasm of patients and they gave up halfway. Some patients also mentioned problems related to mobile phone battery and data charges.

*My phone is quite old, and this app keeps lagging. It takes a long time just to open a single page*.

(P18)

*Sometimes the network is not good, it does not respond for a long time, so I just turn it off*.

(P7)

*Using this drains both battery and data, so I don’t dare keep it running all the time*.

(P27)

Theme 4: Reflexive Monitoring
Human–machine dialogue frustration

Some patients expressed dissatisfaction with the human–computer dialogue experience. They noted that AI often “does not understand” what they are saying, or provides feedback that does not fit their own situation, making the dialogue feel mechanical and awkward. Some respondents also felt tired and frustrated by the repetitive nature of the dialogue, which did not foster a strong sense of reliance on the AI.

*Sometimes it just doesn’t understand what I’m saying and gives advice that doesn’t fit my situation at all*.

(P27)

*I feel like it keeps asking the same questions over and over, and after a while, I get too lazy to respond because it doesn’t feel meaningful*.

(P14)

*It sometimes can’t remember what I said before, every time I have to answer again, I have to ask the same question, really tired*.

(P3)

A sense of uncertainty about the future of AI

Some patients expressed that one of the important barriers affecting their long-term trust and compliance was a strong sense of “future uncertainty”. Although they currently consider AI to be helpful in disease management, they have reservations about its continued effectiveness in the future. Some patients worried that as the disease progressed, AI might not be able to keep up with more complex situations.

*It looks good now, but will it become useless later? COPD is five years, ten years, if it stops, all my records will be gone, I will be wasted*.

(P2)

*At first, I was really impressed, but I’m worried that if my condition becomes more complex, it will reach the limits of what it can do*.

(P25)

## 4. Discussion

This study explored the facilitators and barriers to the use of AI agents for disease management in older COPD patients. Overall, most patients had positive attitudes toward AI agents. Support from themselves, their families, HCPs, policies, or countries promotes patient identification with AI and participation in AI. The positive effects generated through participation and reflections, including “relief and social connection”, “new “doctor”–patient relationship and communication”, “get rid of the burden and return to life”, “benefit and value perception and right self-decision”, also contributed to promoting the use of the AI tool. On the other hand, some patients did not accept AI due to privacy, trust, and boundary issues. Some did not want to participate due to technological anxiety and lack of motivation. After practice and reflection, it was found that the use of AI tools may be hindered by problems such as “extra burden”, “limitations of the physical environment”, “human–machine interaction frustration”, and “a sense of uncertainty about the future of AI”.

In the following discussion, the significance of the study’s findings is interpreted in light of their context and the scope of their applicability. Since the sample was drawn from a single hospital and featured limited diversity in gender and educational background, the generalizability of these findings to other settings or populations may be constrained. A more detailed discussion of the study’s limitations will be provided later.

### 4.1. The Role of NPT in Study

The theories exploring the factors influencing new technologies in the medical field mainly include the Technology Acceptance Model (TAM), Diffusion of Innovations Theory (DOI), and NPT. Kang Y et al. [32] pointed out that TAM focuses on “perceived usefulness and perceived ease of use” as the core logic to predict an individual’s acceptance of technology. It may place excessive focus on individual utility at the micro level, making it easy to overlook macro-level structures and systems. Dearing JW et al. [33] proposed that the DOI pays more attention to the innovation dissemination and early adoption at the institutional level, while ignoring the exploration at the individual level. From the perspective of implementation science, the NPT is used to understand and explain the process by which new technologies are implemented, embedded, and integrated into medical institutions [31]. Applying this theory can help systematically explore the influencing factors in the application process of new technologies. Compared with TAM and DOI, the application of NPT can explain individuals’ attitudes and views towards new technologies from the aspects of coherence and cognitive participation. It describes the collective interactions and embeddedness of AI agents within social relations from the perspectives of collective action and reflexive monitoring. NPT can fully explain the entire process of patients using an AI agent from both micro and macro perspectives. The results of this study indicated that the implementation of AI among patients is indeed influenced by multiple dimensions and levels. These factors include patients’ understanding of the significance of technology, the motivation and support during the participation process, the feasibility of actual use, and the perceived value after use. This aligns with NPT’s framework of understanding→participation→execution→evaluation. NPT has been used by Larsson et al. to explore the barriers to AI triage in primary care in Sweden [34]. Future research can consider issues from an integrated multi-theoretical perspective, seeking to combine NPT with other theories to provide more systematic theoretical support for the promotion of technology.

### 4.2. Key Factors Driving the Integration of AI into Patients’ Daily Lives

Research indicates that AI is not merely a technical tool, but rather a significant initiative for transforming the power structures within traditional healthcare systems, promoting harmonious development in family care, and building an age-friendly society. Firstly, AI avoids unequal doctor–patient communication patterns and has the potential to establish a new type of doctor–patient relationship. This novel relationship breaks through the limitations of expression space caused by the brief face-to-face consultation dominated by HCPs in traditional clinical settings. Patients can express themselves freely and candidly when interacting with AI, which makes “doctor”–patient communication more equitable. These findings align with the research of Xu, Z. et al. [35]. Additionally, AI provides patients with more structured and traceable self-information, thereby enhancing the quality of doctor–patient communication. This is also supported by the views of Hindelang, M. et al. [36]. Future scholars should focus more on how AI can better serve as a bridge between patients and doctors. Secondly, within the family system, AI not only takes on the reminder and supervisory roles typically performed by family caregivers, but also enables patients to gain a clearer understanding of their own health status. This alleviates the concerns and care burden of family carers. Future studies should explore how to optimize the design of AI health management within domestic settings. Thirdly, patients in this study expressed that AI’s extraordinary capabilities allowed them to experience a sense of release and social connection. We speculate that AI holds promise as a means to alleviate social isolation and health inequalities. This notion aligns precisely with one of the international themes for older persons in 2025, which is to promote inclusive social and economic opportunities for older persons through measures enabling their full participation in society [37]. Future research should further examine how AI affects older adults across different socioeconomic statuses, living environments, and levels of digital literacy, to prevent technological interventions from unintentionally widening existing inequalities. In addition, although the results of this study show that AI plays a facilitating role in patient disease management, its potential limitations need to be noted. For example, patients may weaken their own decision-making ability by over-relying on algorithms or perceive a dehumanizing experience under technology-dominated management. This suggests that researchers need to understand the role of AI in disease management from a more prudent perspective, focusing on the potential adverse effects that may accompany its application.

### 4.3. Barriers of Coherence Dimension Deserve Attention

In the results of this study, the three barriers of the coherence dimension deserve attention. Firstly, patients perceived AI as a reluctant choice. This is consistent with the findings of Cadario R et al. [38]. Two explanations may account for this perception. On the one hand, interactions between humans encompass nuanced expressions, emotions, empathy, and ethical responsibilities. In contrast, AI simply simulates a real person through data patterns. This leads patients to believe the value of HCPs is irreplaceable. While AI may assume or reshape certain professional responsibilities, it cannot genuinely replicate the emotional connection inherent in the doctor–patient relationship. On the other hand, society recognizes the costs associated with human medical services. It can be visualized as time and economic investments, including educational and training expenses [39]. In contrast, the initial capital expenditure, integration costs, and ongoing maintenance expenses of AI are challenging for patients to quantify [40]. These differences may result in patients perceiving “AI efforts” and “human efforts” as unequal. The research conducted by El Arab R [40] et al. have demonstrated that AI can lower healthcare costs and time by minimizing unnecessary procedures and optimizing resource utilization. However, patients often perceive this as a low-cost service. The second barrier encompasses privacy conflicts and trust deficiency, which are prevalent issues in contemporary studies [41,42,43]. These factors can result in initial resistance from some patients when adopting AI for disease management. The third barrier is patients often struggle to articulate the triangular relationship among AI, physicians, and themselves. Research indicated that patients are more likely to follow doctors’ recommendations when they perceive AI as a tool to assist doctors and improve access to health information and medical service utilization [44,45]. A study examining the attitudes of patients and the general public toward AI revealed that only a minority believe AI should entirely replace physicians or be entirely excluded from use [46]. This perception may also influence intervention outcomes. Overall, as a recognized tool for cost reduction and efficiency enhancement, the significance of AI cannot be overlooked. It has the potential to improve the efficiency of the medical and health service system. Besides, the future trajectory of AI remains uncertain. From the perspective of long-term development, with the continuous progress of technology, AI is bound to break through the current limitations and promote the future medical and health services to a new stage.

### 4.4. Cross-Cultural Perspectives on Older Adults’ AI Experiences

In the Chinese context, factors such as filial piety, hierarchical respect for doctors, and trust in national policy likely influenced participant engagement with AI. These sociocultural dynamics may not generalize to Western or low-resource contexts. This study focuses on the Chinese context, and international studies provide important implications for how sociocultural factors influence older adults’ use of AI. Wong et al. interviewed older adults in Hong Kong, China, and found that most of the older adults recognized the potential benefits of AI [47]. At the same time, technical ease of use, personalized programs, and privacy concerns play an important role in the adoption of AI health technologies by older adults [47]. A hybrid study based on AI chatbots in the United States revealed that the attitudes of older adults towards AI are divided into “active recipients”, “cautious trialists”, and “avoiders” [48]. There are differences in learning styles and social preferences, suggesting that individual differences should be fully considered when designing AI tools [48]. A qualitative study based on the Q method in South Korea classified the subjective perception of older adults using AI nursing virtual humans, and found that the experience of older users with AI could be divided into different types, such as emotional interaction preference, daily conversation preference, use burden, and function-oriented [49]. This evidence suggests that the experience of AI in older adults is affected by multiple factors such as cultural background, social structure, and technical literacy. Future research should pay more attention to how social, psychological, and technological factors jointly affect the AI experience of older people in cross-cultural contexts, so as to improve the global applicability and policy reference value of research.

### 4.5. Limitations and Future Prospects

First, the sample of this study was from a tertiary hospital in Wuxi, and the sample area and institution type were relatively single, which may limit the generalizability of the results. Future studies should expand the geographical scope to different regions and medical institutions to improve the representativeness of the study conclusions. Second, this study only carried out qualitative discussion from the perspective of patients, which may limit the comprehensive understanding of the research topic to some extent. Future studies can incorporate views from HCPs and caregivers and adopt mixed methods to generate more in-depth and comprehensive evidence.

## 5. Conclusions

This study explored the facilitators and barriers in the process of using AI agents for disease management in older COPD patients, resulting in four themes and nine subthemes, respectively. These findings suggest that the future development of AI should not only focus on achieving higher intelligence and human-like abilities; it must also address system-level issues such as safety, ethics, and cultural acceptance. In addition, to promote the continuous application of AI in patient disease management, it is necessary to simplify the design of the human–computer interface in practice, provide personalized AI literacy training for older adults, and establish a timely feedback mechanism to improve their experience and long-term adherence.

## Figures and Tables

**Table 1:** Mapping of interview outline to theoretical dimensions.

Theoretical Dimension	Question
Coherence	What is your understanding of AI? What do you think is the difference between the use of AI for chronic disease management and other rehabilitation methods? Which approach do you prefer? Why?
Cognitive participation	● What made you decide to join this management model? During the implementation of the project, what makes you want to stick to it? What might prevent you from joining? ● What obstacles have you encountered in the process of disease management using AI agents? How did you overcome these obstacles?
Collective action	How did you feel about your relationships with your health care providers, family, etc., when implementing this model? Can you give me an example? How do you feel about this change?
Reflexive monitoring	● Do you think you have the ability to implement this project well? Is this project easy for you to participate in and implement in the long term? What can be improved? ● What are your experiences and feelings during the whole process of using AI to carry out chronic disease management? What are your views on the use of AI agents to manage disease, and how has this model affected you?

**Table 2:** Participants’ characteristics.

Characteristics	Mean (SD)/*n* (%)
Age	74.04 (5.13)
Gender	
Male	25 (89.29)
Female	3 (10.71)
Education	
Primary school and below	12 (42.86)
Junior high school	15 (53.57)
College or above	1 (3.57)
Marital status	
Never married or divorced or widowed	5 (17.86)
Married	23 (82.14)
Monthly income, RMB, ¥	
<5000	20 (71.43)
≥5000	8 (28.57)
Smoking status	
Still smoking	4 (14.29)
Quit smoking	17 (60.71)
Never smoked	7 (25)
Disease duration, years	
<5	5 (17.86)
5–10	16 (57.14)
>10	7 (25)
GOLD classification	
GOLD Ⅱ	12 (45.86)
GOLD Ⅲ	9 (32.14)
GOLD Ⅳ	7 (25)
Number of hospitalizations in the past year	
None	12 (42.86)
One time	12 (42.86)
Two times or more	4 (14.28)

**Table 3:** Classification of themes, subthemes, and representative quotations for facilitators.

Theme	Subthemes	Representative Quotation
Coherence	Recognition of intelligent technology	Didn’t the TV say we already have smart hospitals? I think that’s pretty great. (P19)
	Supported by policy discourse and the background of national-level projects	Nowadays, the country is vigorously developing and promoting AI, and I feel particularly confident. (P20)
Cognitive Participation	The creation of a family atmosphere	My kids pushed me to try it and helped me download Deepseek on my phone. (P2)
	Recommendations from HCPs	The nurse in charge of me said I could keep using this tool, so I figured it must be legitimate and reliable. (P6)
Collective Action	Relief and social connection	... I’ve come to realize that I’m just a drop in the ocean. (P20)
	New “doctor”–patient relationship and communication	I never thought that doctor-patient communication could be like this... (P11)
	Eliminate the burden and return to life	It reassures her, and it puts my mind at ease, too. (P16)
Reflexive Monitoring	Benefit and value perception	Now I can go out and buy food, and I have something to look forward to. (P11)
	Right self-decision by AI	The growth that AI has brought to me is the most powerful proof of my decision. (P23)

**Table 4:** Classification of themes, subthemes, and representative quotation for barriers.

Theme	Subthemes	Representative Quotation
Coherence	Privacy conflicts and trust deficiency	I think it’s probably just another gimmick for a hospital or a tech company. (P14)
	Blurred boundaries of human–machine responsibility and authority	I can’t tell whether it is my assistant or my leader. (P24)
	Non-high-quality services are chosen reluctantly	Machine service is a helpless choice. (P8)
Cognitive Participation	Technical anxiety	I’m afraid what if I make a mistake. (P13)
	Lack of motivation for continued engagement	I have to do housework and accompany my children every day, and I really can’t stick to using AI. (P12)
Collective Action	Extra burden	Every time you open it, you have to verify, log in, etc., which is too complicated. (P14)
	Limitations of the physical environment	Sometimes the network is not good, and it will not respond for a long time (P7)
Reflexive Monitoring	Human–machine dialogue frustration	Sometimes it doesn’t understand what I’m saying. (P27)
	A sense of uncertainty about the future of AI	It looks good now, but will it become useless later? (P2)

## Data Availability

This study is based on qualitative research, including in-depth interviews and narrative responses. Although all participants provided informed consent, the raw qualitative data contain potentially identifiable and sensitive information that cannot be fully anonymized without compromising the integrity and contextual meaning of the data. Therefore, to protect participant privacy and comply with ethical approval requirements, the full dataset cannot be publicly shared. Nevertheless, the data may be made available upon reasonable request to the corresponding author, subject to ethical considerations and approval, and solely for research purposes. Based on these considerations, we believe that the statement “Dataset available on request from the authors” is accurate and justified.

## Supplementary Materials

The following supporting information can be downloaded at: https://www.mdpi.com/article/10.3390/healthcare14020268/s1, File S1: COREQ Checklist; File S2: Code and theme analysis table (example).
