# Supplementary material for: Facilitators and Barriers of Using an Artificial Intelligence Agent in Chronic Disease Management: A Normalization Process Theory-Guided Qualitative Study of Older Patients with COPD
Source: Healthcare (Basel). 2026 Jan 21;14(2):268. doi: 10.3390/healthcare14020268 (PMC12841200; doi:10.3390/healthcare14020268)
Supplement: Supplementary file 1 [file healthcare-14-00268-s001.zip › Supplementary Materials S1.pdf]

Consolidated criteria for reporting qualitative studies (COREQ): 32-item checklist

Developed from:

Tong A, Sainsbury P, Craig J. Consolidated criteria for reporting qualitative research (COREQ): a 32-item checklist for interviews and focus groups. Int J Qual Health Care. 2007 Dec;19(6):349-57.

No. Item	Guide questions/description	Reported on Page #
Domain 1: Research team and reflexivity		
Personal Characteristics		
1. Interviewer/facilitator	Which author/s conducted the interview or focus group?	4
2. Credentials	What were the researcher's credentials? E.g. PhD, MD	1
3. Occupation	What was their occupation at the time of the study?	1
4. Gender	Was the researcher male or female?	1
5. Experience and training	What experience or training did the researcher have?	4
Relationship with participants		
6. Relationship established	Was a relationship established prior to study commencement?	4
7. Participant knowledge of the interviewer	What did the participants know about the researcher? *e.g. personal goals, reasons for doing the research*	4
8. Interviewer characteristics	What characteristics were reported about the interviewer/facilitator? e.g. Bias, assumptions, reasons and interests in the research topic	4
Domain 2: study design		
Theoretical framework		
9. Methodological orientation and Theory	What methodological orientation was stated to underpin the study? e.g. grounded theory, discourse analysis, ethnography, phenomenology, content analysis	3-4
Participant selection		
10. Sampling	How were participants selected? e.g. purposive, convenience, consecutive, snowball	3-4

11. Method of approach	How were participants approached? e.g. face-to-face, telephone, mail, email	4
12. Sample size	How many participants were in the study?	4
13. Non-participation	How many people refused to participate or dropped out? Reasons?	4
Setting		
14. Setting of data collection	Where was the data collected? e.g. home, clinic, workplace	4
15.Presence of non-participants	Was anyone else present besides the participants and researchers?	4
16. Description of sample	What are the important characteristics of the sample? e.g. demographic data, date	5
Data collection		
17. Interview guide	Were questions, prompts, guides provided by the authors? Was it pilot tested?	3-4
18. Repeat interviews	Were repeat interviews carried out? If yes, how many?	4-5
19. Audio/visual recording	Did the research use audio or visual recording to collect the data?	4-5
20. Field notes	Were field notes made during and/or after the interview or focus group?	4-5
21. Duration	What was the duration of the interviews or focus group?	4
22. Data saturation	Was data saturation discussed?	4
23. Transcripts returned	Were transcripts returned to participants for comment and/or correction?	4-5
Domain 3: analysis and findings		
Data analysis		
24. Number of data coders	How many data coders coded the data?	4-5
25. Description of the coding tree	Did authors provide a description of the coding tree?	Not reported
26. Derivation of themes	Were themes identified in advance or derived from the data?	4-5

27. Software	What software, if applicable, was used to manage the data?	4
28. Participant checking	Did participants provide feedback on the findings?	4-5
Reporting		
29. Quotations presented	Were participant quotations presented to illustrate the themes / findings? Was each quotation identified? e.g. participant number	6-11
30. Data and findings consistent	Was there consistency between the data presented and the findings?	6-11
31. Clarity of major themes	Were major themes clearly presented in the findings?	6-11
32. Clarity of minor themes	Is there a description of diverse cases or discussion of minor themes?	6-11
