# Supplementary material for: Facilitators and Barriers of Using an Artificial Intelligence Agent in Chronic Disease Management: A Normalization Process Theory-Guided Qualitative Study of Older Patients with COPD
Source: Healthcare (Basel). 2026 Jan 21;14(2):268. doi: 10.3390/healthcare14020268 (PMC12841200; doi:10.3390/healthcare14020268)
Supplement: Supplementary file 1 [file healthcare-14-00268-s001.zip › Supplementary Materials S2.pdf]

Code and theme analysis table (example)

Data Extract from Interviews	Codes	Category	Subtheme	Theme
The health advice provided by AI makes me feel reassured that I don't have to worry about my health all the time. (P4)	The sense of security that comes with health advice	Perceived safety and reassurance		
After using this AI, I used to be able to stay at home, but now I can go out for a stroll and buy food. I feel that I have not insisted on exercise in vain, and I have hope for life. (P11)	Resumption of living activities	Improvement in function		
I used to worry about the improper use of drugs, but now thanks to the AI teaching me the correct way to use it... (P19)	Medication guidance	Healthy behavior norms	Benefit and value perception	Reflexive Monitoring
It always answers my questions and cares about me like my friends and family... Every time I finish talking about my pain with it, my mood is much better, and I feel that the future is full of hope. I think the value of AI in the future is immeasurable. (P23)	Companionship and sharing pain	Psychological support		
It records my physical data and develops targeted rehabilitation goals and rehabilitation strategies, and it helps a lot. (P26)	Development of individualized rehabilitation goals and strategies	Personalized health management		
